# Maternal Dietary Exposure to Low-Dose Bisphenol A Affects Metabolic and Signaling Pathways in the Brain of Rat Fetuses

**DOI:** 10.3390/nu12051448

**Published:** 2020-05-17

**Authors:** Claudia Tonini, Marco Segatto, Simone Gagliardi, Simona Bertoli, Alessandro Leone, Laura Barberio, Maurizio Mandalà, Valentina Pallottini

**Affiliations:** 1 Department of Science, University Roma Tre, Viale Marconi 446, 00146 Rome, Italy; claudia.tonini@uniroma3.it (C.T.); gsimone@hotmail.it (S.G.); 2 Department of Biosciences and Territory, University of Molise, Contrada Fonte Lappone, 86090 Pesche (IS), Italy; marco.segatto@unimol.it; 3 International Center for the Assessment of Nutritional Status (ICANS), Department of Food Environmental and Nutritional Sciences (DeFENS), University of Milan, Via Mangiagalli 25, 20133 Milan, Italy; simona.bertoli@unimi.it (S.B.); alessandro.leone1@unimi.it (A.L.); 4 Istituto Auxologico Italiano, IRCCS, Lab of Nutrition and Obesity Research, 20100 Milan, Italy; 5 Department of Biology, Ecology and Earth Science, University of Calabria, Arcavacata di Rende, 87036 Cosenza (CS), Italy; laura.barberio90@gmail.com (L.B.); m.mandala@unical.it (M.M.)

**Keywords:** bisphenol A, cholesterol, 3-Hydroxy 3-methylglutaryl Coenzyme A reductase, mevalonate pathway, neurotrophins

## Abstract

Bisphenol A (BPA) is a synthetic compound widely used for the production of polycarbonate plasticware and epoxy resins. BPA exposure is widespread and more than 90% of individuals have detectable amounts of the molecule in their body fluids, which originates primarily from diet. Here, we investigated whether prenatal exposure to BPA affects the mevalonate (MVA) pathway in rat brain fetuses, and whether potential effects are sex-dependent. The MVA pathway is important for brain development and function. Our results demonstrate that the fetal brain, exposed in utero to a very low dose of BPA (2.5 µg/kg/day), displayed altered MVA pathway activation, increased protein prenylation, and a decreased level of pro-BDNF. Interestingly, the BPA-induced effects on estrogen receptor α were sex-dependent. In conclusion, this work demonstrates intergenerational effects of BPA on the brain at very low doses. Our results reveal new targets for BPA-induced interference and underline the impacts of BPA on health.

## 1. Introduction

Bisphenol A (BPA) is a synthetic compound that is widely used to produce polycarbonate plastic and epoxy resins [1]. It is present in many consumer products including food and beverage containers, thermal receipt paper, medical devices, cosmetics, and toys. In industrialized countries, BPA exposure is widespread and more than 90% of individuals have detectable amount of BPA in their body fluids, with exposure resulting primarily from diet [2]. BPA is a widely recognized endocrine disruptor that interferes with estrogen and androgen receptor (ER and AR respectively) function, as well as with thyroid hormone receptors (TR) [3]. BPA has a plethora of dangerous effects on human health, which are tissue-specific and depend on onset, dose, and duration of exposure [3]. In this context, developing fetuses are especially vulnerable to BPA exposure, even at very low doses. Considerable levels of BPA are detected in placenta tissue, amniotic fluid, and fetal plasma. This is alarming since it is well established that BPA is able to cross the placenta and the blood brain barrier (BBB), thus affecting fetal brain development and function [4,5]. Studies performed on primary cell cultures from prenatally exposed rodents revealed that BPA reduces the proliferation of pluripotent neural progenitors and alters the neuronal/glial progenitor ratio [6,7]. Similarly, several studies reported the disruption of neurogenesis and neuronal migration in the embryonic mouse brain after maternal exposure [8,9]. Moreover, BPA may have a powerful influence on dimorphic brain differentiation mainly through its well-characterized estrogenic [3] and antiandrogenic [3,10] activities. Among their multiple functions, estrogens modulate cholesterol homeostasis [11,12,13]. Accordingly, there is increasing evidence that BPA affects hepatic cholesterol levels by altering the expression of proteins involved in cholesterol biosynthesis, possibly through the activation of estrogen receptors [14].

Cholesterol biosynthesis starting with the mevalonate (MVA) pathway plays a key role in the brain, and its dysfunction has been related to neurodevelopmental disorders [15,16]. Cholesterol has different structural roles in the brain: it is an important constituent of neuronal membranes and a major component of the myelin sheath surrounding axons [17]. Notably, cholesterol is essential for the formation, shape, and release of synaptic vesicles and determines the organization and positioning of neurotransmitter receptors [18,19]. Besides cholesterol, other MVA pathway end-products play essential roles in the brain. For instance, prenyls are required for the post-translational modification of small GTPases, which are involved in crucial neurobiological functions. Protein prenylation involves the covalent binding of farnesyl pyrophosphate (FPP) or geranylgeranyl pyrophosphate (GGPP) moieties to proteins, allowing their translocation to cell membranes and, in turn, the activation of key signaling cascades [20,21]. Ras proteins, a family of farnesylated proteins, mediate a plethora of signal transduction pathways controlling cell proliferation and differentiation. Moreover, Ras modulates processes related to learning, memory and behavioral responses that involve rearrangements of synaptic connectivity [20,21,22]. RhoA is a geranylgeranylated G protein that controls gene transcription and actin cytoskeleton remodeling and regulates neurite outgrowth and synaptic connectivity [23,24]. Furthermore, active RhoA suppresses the activation of cAMP responsive element binding protein 1 (CREB1). This key transcription factor controls memory, neurogenesis, neuron survival and trophism [20], as it regulates levels of neurotrophic factors, such as brain-derived neurotrophic factor (BDNF) and nerve growth factor (NGF) [25,26].

The key and rate-limiting enzyme of the MVA pathway is the 3-hydroxy-3-methylglutaryl CoA reductase (HMGCR) [21]. Its activity is tightly regulated. Short-term regulation is based on phosphorylation and de-phosphorylation, mainly through AMP activated kinase (AMPK) and protein phosphatase 2A (PP2A), respectively [27,28]. Long-term regulation affects the protein levels through transcription and degradation [29]. Transcription is controlled by the sterol regulatory element binding protein 2 (SREBP2), which responds to changes in intracellular sterol level as part of a negative feedback mechanism [30]. Apart from enzymes involved in cholesterol biosynthesis, SREBP2 also controls the transcription of genes encoding the proteins involved in cholesterol transport and uptake, such as low-density lipoprotein receptor (LDLr) [29,30,31].

Despite the importance of the MVA pathway and cholesterol homeostasis for brain development and function, the effects of prenatal exposure to BPA on these key metabolic processes have not been studied. Our study reveals specific intergenerational and sex-dependent defects in the fetal brain induced by low doses of BPA.

## 2. Materials and Methods

### 2.1. Animals

All experiments were carried out in accordance with the European Guidelines for the care and use of laboratory animals (Directive 26/2014/EU), and they were approved by the local ethical committee of the University of Calabria and by the Italian Ministry of Health (license n.74/2018-PR). Experiments were performed on female Sprague Dawley rats at 8 weeks of age. Rats were divided into four groups treated with BPA (Sigma Aldrich, Milan, Italy) at 2.5 µg/kg/day or 25 µg/kg/day or 250 µg/kg/day, or BPA vehicle. BPA was administered to rats via drinking water. The factual intake was determined based on the daily difference of drinking water volume and body weight gain. Control rats were treated with 0.05% ethanol (BPA vehicle) corresponding to the highest amount of ethanol present in the drink water of our rats treated with BPA. BPA or ethanol were administered for one month (virgin state) plus 20 days during pregnancy. A female in proestrus was placed with a fertile male overnight. A vaginal smear to detect spermatozoa was performed the following morning to confirm day 1 of pregnancy. All rats were housed individually in the animal care facility, maintained under controlled conditions on a 12-h light/dark photoperiod and provided commercial chow and tap water ad libitum.

Animals were euthanized on the 20th day of pregnancy with isoflurane inhalation, and the uterus was removed. Blood was collected in 1 mL tubes and serum was allowed to clot at room temperature for 30 min, then centrifuged at 2000× *g* for 10 min, at 4 °C. After the centrifugation, the serum was carefully collected for the following analyses.

Fetuses removed from the uterus were collected in a beaker containing physiological solution, HEPES-PSS, at 4 °C. The first two fetuses starting from the cervix end of one horn were used for our analyses. The fetuses were placed in Petri dishes containing HEPES-PSS (4 °C) to determine the gender and to isolate the brain. The sex of the fetus was determined on the base of the ano-urethra distance evaluated using a stereomicroscope. In males, the distance between anus and urethra is about 1.5–2 times longer than in females. In addition, we confirmed the sex by examination of the genitals. Fetal brains were dissected immediately after decapitation, the skull was gently opened by surgical scissors and the brain was quickly removed and stored at −80 °C.

### 2.2. Cholesterol, HDL, LDL, and Triglyceride Content

Triglycerides and total-, LDL- and HDL-cholesterol were measured by means of an enzymatic method (Cobas Integra 400 Plus, Roche Diagnostics, Rotkreuz, Switzerland), with intra- and inter-assay coefficients of variation < 2%. The cholesterol amount in brain tissue was quantified by the Cholesterol Quantitation Kit-MAK043 (Sigma Aldrich, Milan, Italy) following the manufacturer’s instructions.

### 2.3. Total Lysate and Membranes Preparation for Western Blot Analysis

Brain samples were lysed in 1:5 *w/v* homogenization buffer (Sucrose 0.1 M, KCl 0.05 M, KH2PO4 0.04 M, EDTA 0.04 M, pH 7.4, with 1:1000 protease inhibitor cocktail and 1:400 phosphatase inhibitor cocktail, Sigma-Aldrich) by sonication (VCX 130 PB, Sonics, Newtown, 06,470 CT) on ice, for 30 s, and centrifuged at 10,000 rpm for 10 min, at 4 °C to yield total lysate. To isolate membrane fractions, total lysate was centrifuged at 14,000 rpm for 1 h at 4 °C and the pellet was solubilized in homogenization buffer by sonication. Protein concentration was assessed by the method of Lowry [32]. Aliquots of homogenate samples were diluted with Laemmli buffer, boiled for 5 min and subjected to sodium dodecyl sulfate polyacrylamide gel electrophoresis (SDS-PAGE) for subsequent Western blot analysis.

### 2.4. Immunoblotting

Proteins (15 µg) from total lysates were separated by SDS-PAGE at 50 mA (constant current) first, and then at 120 V for 120 min. Subsequently, proteins were transferred to nitrocellulose membranes through Trans-Blot Turbo Transfer System (Bio-Rad Laboratories). The nitrocellulose membrane was incubated at room temperature with 5% fat-free milk in Tris-buffered saline (NaCl 0.138 M, KCl 0.027 M, Tris-HCl 0.025 M, and 0.05% Tween-20, pH 6.8), and then overnight, at 4 °C, with primary antibody, followed by 1 h of incubation with secondary peroxidase-conjugated antibody produced in mouse or in rabbit (1:10,000; Biorad). Immunoreactivity was detected using the clarity ECL Western blotting system (Bio-Rad Laboratories) and a ChemiDoc MP system (Bio-Rad Laboratories). The following primary antibodies were tested: HMGCR (Abcam, ab242315, dilution 1:1000), P-HMGCR Ser872 (Merck Millipore, #09-356, dilution 1:1000), P-AMPK Thr172 (Sigma-Aldrich, #15-115, dilution 1:1000), RhoA (Santa Cruz Biotechnology, Santa Cruz, CA, USA, sc-418, dilution 1:500); Ras (Santa Cruz Biotechnology, sc-53959, dilution 1:500), P-CREB-1 Ser133 (Santa Cruz Biotechnology, sc-7978, dilution 1:1000), LDLr (Abcam, ab30532, dilution 1:1000), nSREBP2 (Abcam, ab30682, dilution 1:1000), BDNF (Santa Cruz Biotechnology, sc-546, dilution 1:1000), NGF (Santa Cruz Biotechnology, sc-365944, dilution 1:2000), P-Akt Ser473 (Cell Signalling, 193H12, dilution 1:500), ERα (D12) (Santa Cruz Biotechnology, sc-8005, dilution 1:1000), P-ERα Ser118 (Cell Signalling, 16J4, dilution 1:1000). As loading control, the immunoblots were reacted with an antibody against tubulin, actin, or vinculin (1:10,000; Sigma Aldrich, Milan, Italy). Western blot images were analyzed by ImageJ (National Institutes of Health, Bethesda, MD, USA) software for Windows. Intensities of proteins of interest were normalized to intensities of respective housekeeping proteins.

### 2.5. Statistical Analyses

Data are expressed as mean ± SD for all experiments. The experiments were performed in duplicate (technical replicates) and at least six animals per experimental group were used (biological replicates). Statistically significant differences were tested by one-way ANOVA for dams’ weight and plasma lipids and by two-way analysis of variance (ANOVA), followed by Bonferroni post-hoc test for all the other tests (*p* < 0.05 level). Statistical analyses and graph editing were performed using GRAPHPAD Prism6 (GraphPad, La Jolla, CA, USA) for Windows.

## 3. Results

**Figure 1:**
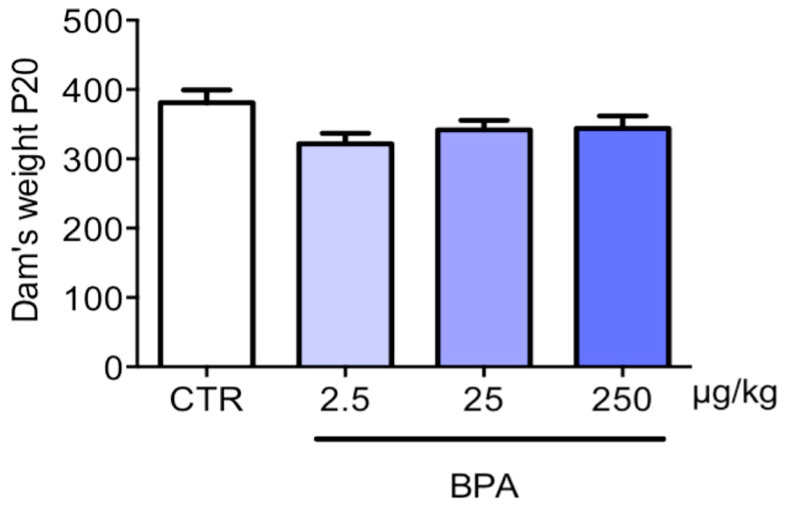
Dams’ weight after BPA exposure. Pregnant rats were exposed to three different doses of BPA (2.5- 25- 250 µg/kg/day) or vehicle (CTR) via drinking water, starting 30 days before the beginning of pregnancy until their sacrifice at GD 20. Weights were measured at the end of the experiments. Statistical analysis was carried out by one-way ANOVA. *n* = 7 for each experimental group.

To uncover direct effects of BPA on the brain, we first tested whether the substance affected metabolic parameters of the dams which could impair embryo development indirectly. BPA had no effect on the weight of pregnant rats (Figure 1) or their plasma lipid profiles (Table 1).

**Figure 2:**
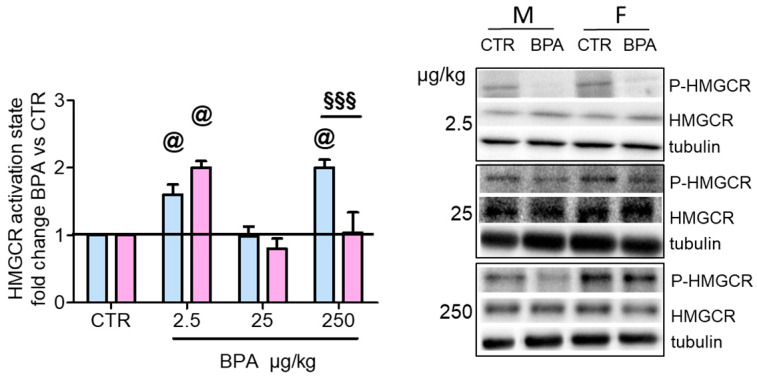
Activation state of HMGCR in the brain of rat fetuses in utero exposed to different doses of BPA. On the left, HMGCR activation state in male (M, blue) and female (F, pink) fetuses of dams exposed to three different doses of BPA (2.5- 25- 250 µg/kg/day) The substance was administered through drinking water starting 30 days before the beginning of pregnancy until their sacrifice at GD 20. HMGCR activation state was estimated by the ratio of total and phosphorylated form of the enzyme and normalized to the values of the vehicle-treated animals. Tubulin served as housekeeping protein to correct for protein loading. *n* = 6 for each experimental group. Images on the right show representative Western blots. Statistical analysis was carried out by two-way ANOVA followed by Bonferroni post-hoc test among the sample treated with the same dose. @ = *p* < 0.001 vs. sex-matched CTR; §§§ = *p* < 0.001 between male and female.

Subsequently, we examined whether prenatal exposure to BPA affected the MVA pathway in the brain of male and female rat fetuses. The ratio between the total amount of HMGCR and of its phosphorylated form reflects the activation state of the enzyme in pathophysiological contexts [24]. Prenatal exposure to BPA modulated HMGCR activation in a dose- and sex-dependent manner. No differences between sexes were observed in control animals. Specifically, BPA significantly increased HMGCR activation at the lowest dose (2.5 µg/kg/day) in both male and female fetuses. Differently, a medium dose (25 µg/kg/day) showed no effect whereas the highest dose tested (250 µg/kg/day) affected only males, but not females (Figure 2). These results are in agreement with the well-established notions that endocrine disruptors induce biological effects by U-shaped dose–response curves [3] and that, sometimes, males but not females show non-monotonic dose–response curves in certain tests [33].

**Figure 3:**
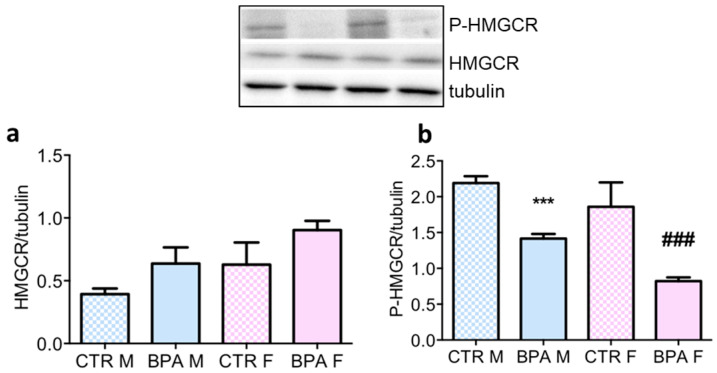
HMGCR total and phosphorylation levels in the brain of rat fetuses in utero exposed to 2.5 µg/kg/day of BPA. Representative western blot and densitometric analysis of total HMGCR (**a**) and P-HMGCR (**b**). *n* = 6 for each experimental group. Tubulin served as housekeeping protein to normalize protein loading. Statistical analysis was carried out by using two-way ANOVA followed by Bonferroni post-hoc test. *** = *p* < 0.001 vs. CTR M; ### = *p* < 0.001 vs. CTR F.

At present, it is unclear whether exposure to low-dose BPA is detrimental to human health, especially when it is below the tolerable daily intake (4 µg/kg/day) indicated by the European Food Safety Authority (EFSA). To address this topic, all following experiments were performed with 2.5 µg/kg/day BPA. As shown in Figure 3a and b respectively, the activation state of HMGCR did not depend on alteration of its levels but was induced by a decrease in its phosphorylation [30].

**Figure 4:**
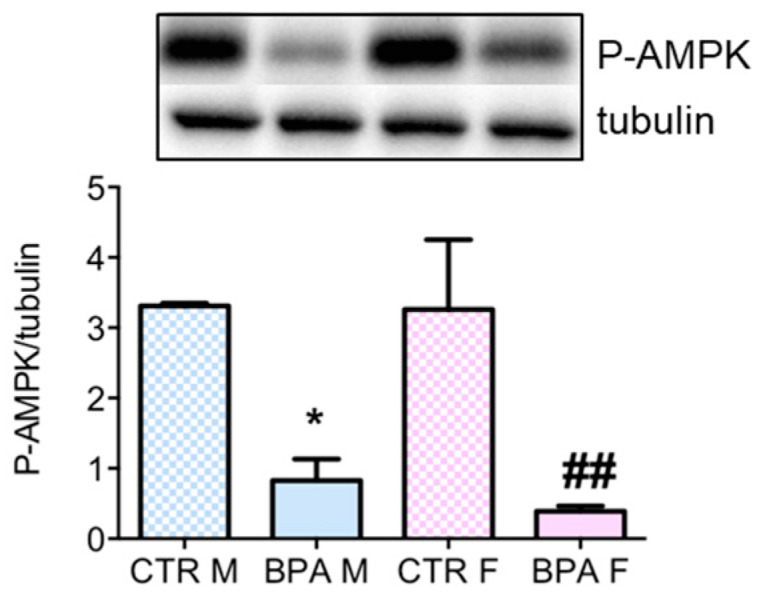
AMPK phosphorylation in the brain of rat fetuses in utero exposed to 2.5 µg/kg/day of BPA. Representative western blot and densitometric analysis of P-AMPK. Tubulin served as housekeeping protein to normalize protein loading. *n* = 6 for each experimental group. Statistical analysis was carried out by using two-way ANOVA followed by Bonferroni post-hoc test. * = *p* < 0.05 vs. CTR M; ## = *p* < 0.01 vs. CTR F.

Based on these results, we evaluated the involvement of AMPK, which is the main upstream kinase regulating HMGCR. As AMPK activation depends on the phosphorylation of its catalytic subunit [27], so the phosphorylation state of AMPK was analyzed. As shown in Figure 4, BPA exposure suppressed AMPK phosphorylation in both male and female fetuses, which fits well with the low P-HMGCR levels detected in exposed fetuses.

**Figure 5:**
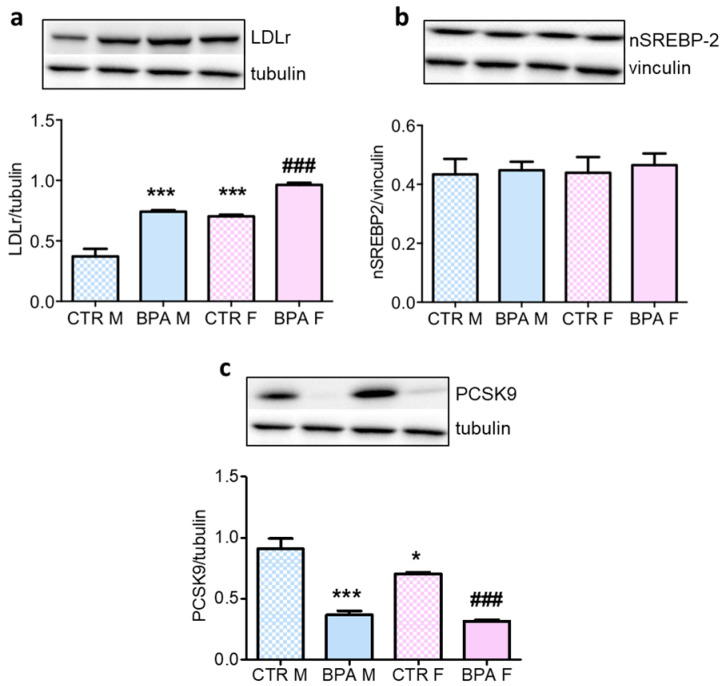
LDLr, nSREBP2 and PCSK9 content in the brain of rat fetuses in utero exposed to 2.5 µg/kg/day of BPA. Representative Western blot and densitometric analysis of LDLr (**a**), nSREBP2 (**b**) and PCSK9 levels (**c**). *n* = 6 for each experimental group. Tubulin or vinculin served as housekeeping proteins. Statistical analysis was carried out by using two-way ANOVA followed by Bonferroni post-hoc test. * = *p* < 0.05 vs. CTR M; *** = *p* < 0.001 vs. CTR M; ### = *p* < 0.001 vs. CTR F.

Cholesterol homeostasis is strictly based on an adequate balance between biosynthesis and extracellular uptake. The latter process is mainly operated by LDLr, which mediates the internalization of LDL through receptor-mediated endocytosis [17,29]. Our results demonstrate that the LDLr level was higher in female fetuses compared to males under normal conditions (Figure 5a). BPA exposure increased its levels in both male and female fetuses. To uncover the mechanisms underlying this change, both the negative post-translational regulator of LDLr, PCSK9 [34] and its transcription factor (the nuclear active fragment of SREBP2 (nSREBP2)) were analyzed. BPA exposure reduced the level of PCSK9 (Figure 5c) without affecting the level of nSREBP2 (Figure 5b), which is in also good agreement with the unmodified levels of HMGCR which is also a SREBP2 target gene. Notably, the modulation of PCSK9 occurred in a sex-dependent manner similar to LDLr.

Having analyzed the principal proteins involved in cholesterol synthesis and uptake, we then measured downstream products of the MVA pathway. Our results show that the cholesterol content in the brain of rat fetuses was not affected by BPA prenatal exposure (Table 2).

**Figure 6:**
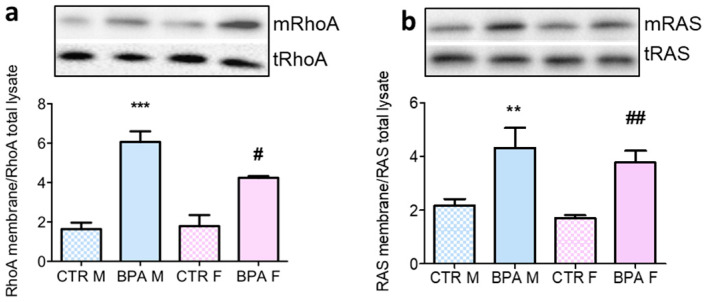
RhoA and Ras activation in the brain of rat fetuses in utero exposed to 2.5 µg/kg/day of BPA. Representative Western blots of membrane-bound RhoA (**a**) and Ras (**b**) and their content in total lysate of fetuses brain from prenatally BPA exposed and CTR animals. The graphs represent the ratio of membrane-bound and total levels of the proteins in brain lysates. *n* = 6 for each experimental group. Statistical analysis was carried out by using two-way ANOVA followed by Bonferroni post-hoc test. ** = *p* < 0.01 vs. CTR M; *** = *p* < 0.001 vs. CTR M; # = *p* < 0.05 vs. CTR F; ## = *p* < 0.01 vs. CTR F.

Besides cholesterol, the MVA pathway also produces isoprenoid intermediates (FPP and GGPP), which regulate the subcellular location and activity of intracellular small G-proteins [20]. As these key molecular switches orchestrate neuronal survival and plasticity, activated membrane-bound RhoA and Ras were analyzed as prototypes of geranyl-geranylated and farnesylated proteins, respectively. BPA enhanced both RhoA and Ras membrane translocation, which is in full agreements with the enhanced HMGCR activity (Figure 6).

**Figure 7:**
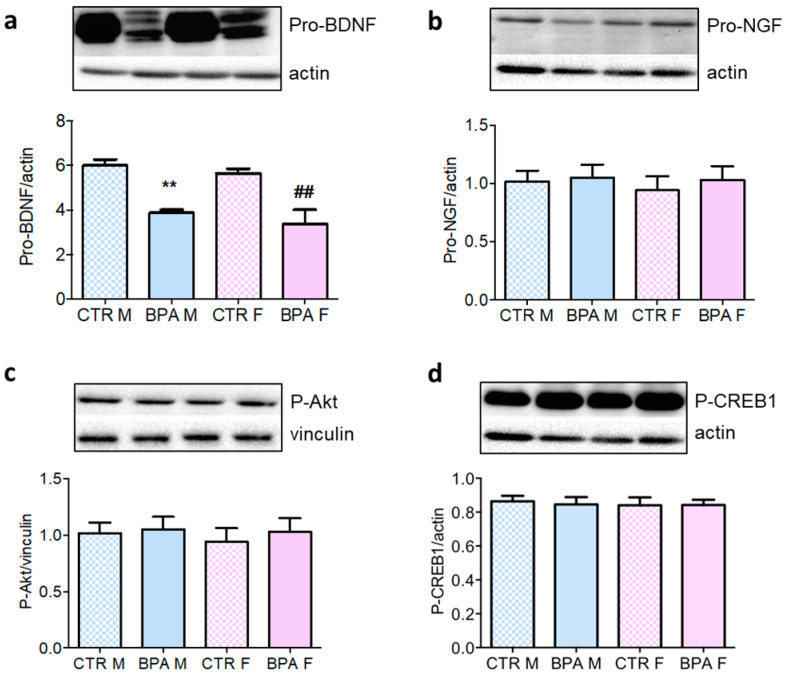
Pro-BDNF, Pro-NGF content, Akt and CREB1 phosphorylation in the brain of rat fetuses in utero exposed to 2.5 µg/kg/day of BPA. Representative Western blot and densitometric analysis of pro-BDNF (**a**) and pro-NGF levels (**b**), CREB-1 (**c**) and Akt phosphorylation (**d**) performed on brain lysates from prenatally exposed to BPA and CTR animals. *n* = 6 for each experimental group. Vinculin or actin served as housekeeping proteins. Statistical analysis was carried out by using two-way ANOVA followed by Bonferroni post-hoc test. ** = *p* < 0.01 vs. CTR M; ## = *p* < 0.01 vs. CTR F.

A key signal regulating neuronal development is BDNF [20]. Previous studies revealed that a high dose of BPA (200 µg/kg/day) affects BDNF gene expression [35] which may compromise brain development. We observed that even a low dose of this compound was sufficient to reduce the level of pro-BDNF by 30% (Figure 7a), while no alterations were observed in the amount of pro-NGF (Figure 7b). Remarkably, these changes occurred in male and female fetuses. CREB1 represents one of the main transcription factors that regulates the expression of neurotrophin [20,36,37]. However, our analysis revealed no effect of BPA on the phosphorylation status of Akt and CREB1 following prenatal exposure (Figure 7c,d).

**Figure 8:**
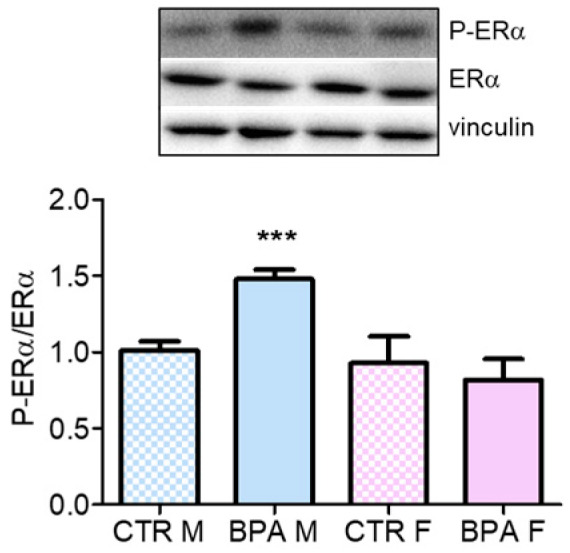
ERα phosphorylation state in the brain of rat fetuses in utero exposed to 2.5 µg/kg/day of BPA. Representative western blot of P-ERα and total ERα. The graph represents the densitometric analysis of the ratio between phosphorylated and total form. Data are expressed as means ± SD. Vinculin served as housekeeping protein to normalize protein loading. Statistical analysis was carried out by using two-way ANOVA followed by Bonferroni post-hoc test. *** = *p* < 0.001 vs. CTR M.

We next tested whether BPA exerts its effects by disrupting ER signaling, which is the classic mode of action of this compound and since it has been demonstrated that estrogens are able to modulate cholesterol homeostasis. Interestingly, prenatal exposure to low doses of BPA exerted a sex-dependent response by exclusively affecting male fetuses (Figure 8), suggesting that sex-independent effects were mediated by distinct pathways.

## 4. Discussion

According to the hypothesis of the fetal origin of adult diseases, stressors derived from the mother “permanently change the body’s structure and function in ways which program the appearance of disease in later life” [38]. In fact, prenatal development is a critical window of elevated vulnerability, where the individual is particularly sensitive to environmental stressors. Notably, the brain represents one of the most vulnerable organs in our body. A hallmark of brain development is the high capacity of adaptation through the continuous adjustment of neuron number, balancing neurogenesis and apoptosis, and neuronal connectivity, through the formation and elimination of synapses. These processes operate within a remarkably complex and highly dynamic network, which is guided by genetic and epigenetic signals and adapts to changes in the environment. On the other hand, this plasticity renders the brain structure highly vulnerable to external environmental factors, including diet, maternal behavior, and xenocompounds [39]. Several reports highlighted that cholesterol homeostasis is required for the correct development and function of the nervous system [19,21,22]. Consequently, alterations in this metabolic pathway during fetal development may contribute to specific pathological conditions, including neurodevelopmental and neurodegenerative diseases [15,16,18,40]. In the present work, we studied the effects of a low dose of BPA on the MVA pathway in fetal rat brains, but also other key molecules that can be involved in brain development. Notably, the dose we used is below the threshold indicated by EFSA (4 µg/kg/day). Our results demonstrate that BPA induces HMGCR activation and enhances LDLr expression without any change in total cholesterol content. However, we cannot exclude local or cell-specific changes. The data could suggest that the BPA-induced effects on MVA pathway are mediated by post-translational mechanisms. Indeed, the expression of the nuclear and transcriptionally active fragment of SREBP2 was unaltered upon BPA prenatal exposure, which may exclude transcriptional changes of HMGCR and LDLr levels. On the other hand, BPA inhibited AMPK phosphorylation, which in turn results in a lower phosphorylation rate of HMGCR. The increase of LDLr levels may be caused by a reduced degradation rate modulated by PCSK9 [34]. The increase of HMGCR activity can also increase the content of other MVA end-products such as prenyls and may enhance the level of protein prenylation. This was indicated by our observation that BPA prenatal exposure significantly increased the amount of membrane-associated RhoA and RAS. These small GTPases are involved in numerous signal transduction pathway whose balance between activation/inactivation allows the central nervous system to develop and function properly. For instance, RAS participates in the control of cell proliferation and morphology, as well as synaptic connectivity mechanisms [20,22]. Similarly, RhoA supports integrating extracellular and intracellular signals to coordinate actin cytoskeleton remodeling. Several reports highlight the critical role of MVA pathway and RhoA in neurite formation and synaptic connectivity [24,27]. Notably, RhoA activity has been related to neurite retraction [41], whereas its decrease has been associated to neurite elongation [23,24]. Moreover, other researches demonstrate that prenylation is increased in several neurological diseases [42]. So, BPA-induced alterations of this key regulatory proteins could perturb brain development and functions.

Our results also demonstrate that BPA reduced the levels of neurotrophin precursor pro-BDNF. BDNF and NGF are synthetized as precursors (pro-BDNF and pro-NGF respectively), which represent the predominant neurotrophin forms present in different neuronal tissues [43,44]. BPA may lower *bdnf* gene transcription through epigenetic deregulation. It is known that this endocrine disruptor affects DNA methylation of transcriptionally relevant region of *bdnf* gene [35]. This hypothesis is supported by previous findings that BPA treatment significantly decreases BDNF mRNA levels [45]. Interestingly, BPA did not affect the Akt/CREB1 axis in our experimental context suggesting that BPA-induced modulation of pro-BDNF levels is independent from this transduction pathway and, possibly, from MVA pathway. However, it cannot be excluded that pro-BDNF changes may be associated to other MVA pathway-dependent signaling cascades that are not evaluated in this work. The different behavior of pro-BDNF and pro-NGF expression suggests independent mechanisms of regulation mediated by BPA exposure. Our results support other studies, as the expression of these neurotrophins can be subjected to diverse regulations by the same stimuli [46,47,48]. Finally, as BPA is primary known as endocrine disruptor, we evaluated its impact on ERα phosphorylation. The phosphorylation on Ser118 has profound effects on receptor activity, as it influences the recruitment of co-activators that enhance ER-mediated gene transcription [49,50]. Interestingly, BPA prenatal exposition affected ERα phosphorylation in male but not in female fetuses. This result suggests that males are more vulnerable to this endocrine disruptor than females [33]. The effect of BPA on P-ERα levels is not unexpected since BPA is structurally similar to estradiol, and thus it is able to bind ERα, inducing its phosphorylation and configuration change [3], the reason why female seems to be less vulnerable in this context needs further investigations. Moreover, previous reports indicated that BPA-dependent estrogenic activity flows through the small pool of ERα localized at the plasma membrane which mediates rapid signal transduction events [51,52]; thus, the post-translational modulation of MVA-related proteins, at least in males, could also depend on the ERα-dependent signaling at the plasma membrane-starting signals.

**Figure 9:**
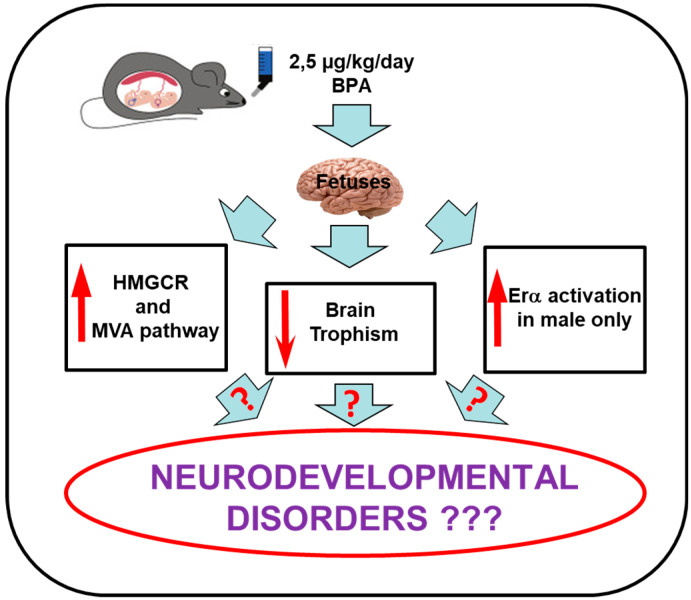
Graphical model summarizing principal results.

Overall, the results presented herein underline that even very low doses of BPA in pregnant drinking water can perturb key metabolic and signaling pathways in a sex-dependent and -independent manner (Figure 9). Notably, these effects occur despite the efficient drug-metabolizing system of dams [4]. The effects of BPA may be due to the efficient placental transfer of both BPA and its glucuronide derivative, coupled to the deficiency of fetal UDP glucuronosyltransferase (UGT) enzyme, which eliminates xenobiotic substances [4]. Combined, these factors permit a remarkable amount of active BPA in the fetal compartment. Subsequently, the substance can easily enter the central nervous system as it can cross the blood-brain barriers [3,5,8]. In conclusion, this work demonstrates an intergenerational effect of a very low dose of BPA on metabolic and signaling pathways that are important for proper brain development and function, thus underlining the potential dangers for human health.

## Figures and Tables

**Table 1:** Plasma lipid content in dams drinking vehicle or BPA.

mg/dl Plasma	CTR Vehicle	BPA 2.5 µg/kg/day	BPA 25 µg/kg/day	BPA 250 µg/kg/day
Cholesterol	88.0 ± 14.4	70.8 ± 17.4	75.6 ± 14.1	83.4 ± 14.2
HDL	61.3 ± 10.9	46.0 ± 12.2	49.4 ± 12.6	59.7 ± 12.7
LDL	16.5 ± 9.4	10.0 ± 2.5	11.9 ± 2.3	12.7 ± 2.4
Triglycerides	299.7 ± 104.9	366.2 ± 142.0	341.4 ± 70.7	342.7 ± 42.5

**Table 2:** Brain cholesterol content in prenatal BPA- or Vehicle-exposed rat fetuses.

	CTR M	BPA M	CTR F	BPA F
Cholesterol (mg/g tissue)	3.53 ± 1.1	4.39 ± 1.4	3.40 ± 1.4	4.80 ± 1.9

## References

[1] Geens T., Goeyens L., Covaci A. (2011). Are potential sources for human exposure to bisphenol-A overlooked?. Int. J. Hyg. Environ. Health.

[2] Geens T., Aerts D., Berthot C., Bourguignon J.P., Goeyens L., Lecomte P., Maghuin-Rogister G., Pironnet A.M., Pussemier L., Scippo M.L. (2012). A review of dietary and non-dietary exposure to bisphenol-A. Food Chem. Toxicol..

[3] Acconcia F., Pallottini V., Marino M. (2015). Molecular Mechanisms of Action of BPA. Dose Response.

[4] Nishikawa M., Iwano H., Yanagisawa R., Koike N., Inoue H., Yokota H. (2010). Placental transfer of conjugated bisphenol A and subsequent reactivation in the rat fetus. Environ. Health Perspect..

[5] Itoh K., Yaoi T., Fushiki S. (2012). Bisphenol A, an endocrine-disrupting chemical, and brain development. Neuropathology.

[6] Kim K., Son T.G., Kim S.J., Kim H.S., Kim T.S., Han S.Y., Lee J. (2007). Suppressive effects of bisphenol A on the proliferation of neural progenitor cells. J. Toxicol. Environ. Health A.

[7] Okada M., Murase K., Makino A., Nakajima M., Kaku T., Furukawa S., Furukawa Y. (2008). Effects of estrogens on proliferation and differentiation of neural stem/progenitor cells. Biomed. Res..

[8] Komada M., Asai Y., Morii M., Matsuki M., Sato M., Nagao T. (2012). Maternal bisphenol A oral dosing relates to the acceleration of neurogenesis in the developing neocortex of mouse fetuses. Toxicology.

[9] Mathisen G.H., Yazdani M., Rakkestad K.E., Aden P.K., Bodin J., Samuelsen M., Nygaard U.C., Goverud I.L., Gaarder M., Løberg E.M. (2013). Prenatal exposure to bisphenol A interferes with the development of cerebellar granule neurons in mice and chicken. Int. J. Dev. Neurosci..

[10] Picot M., Naulé L., Marie-Luce C., Martini M., Raskin K., Grange-Messent V., Franceschini I., Keller M., Mhaouty-Kodja S. (2014). Vulnerability of the neural circuitry underlying sexual behavior to chronic adult exposure to oral bisphenol a in male mice. Endocrinology.

[11] Marino M., Distefano E., Pallottini V., Caporali S., Bruscalupi G., Trentalance A. (2001). Activation of IP(3)-protein kinase C-alpha signal transduction pathway precedes the changes of plasma cholesterol, hepatic lipid metabolism and induction of low-density lipoprotein receptor expression in 17-beta-oestradiol-treated rats. Exp. Physiol..

[12] Segatto M., Trapani L., Marino M., Pallottini V. (2011). Age- and sex-related differences in extra-hepatic low-density lipoprotein receptor. J. Cell. Physiol..

[13] Segatto M., Di Giovanni A., Marino M., Pallottini V. (2013). Analysis of the protein network of cholesterol homeostasis in different brain regions: An age and sex dependent perspective. J. Cell. Physiol..

[14] Li Q., Zhang H., Zou J., Mai H., Su D., Feng X., Feng D. (2019). Bisphenol A exposure induces cholesterol synthesis and hepatic steatosis in C57BL/6 mice by down-regulating the DNA methylation levels of SREBP-2. Food Chem. Toxicol..

[15] Cartocci V., Servadio M., Trezza V., Pallottini V. (2017). Can Cholesterol Metabolism Modulation Affect Brain Function and Behavior?. J. Cell. Physiol..

[16] Segatto M., Tonini C., Pfrieger F.W., Trezza V., Pallottini V. (2019). Loss of Mevalonate/Cholesterol Homeostasis in the Brain: A Focus on Autism Spectrum Disorder and Rett Syndrome. Int. J. Mol. Sci..

[17] Pfrieger F.W., Ungerer N. (2011). Cholesterol metabolism in neurons and astrocytes. Prog. Lipid Res..

[18] Segatto M., Leboffe L., Trapani L., Pallottini V. (2014). Cholesterol homeostasis failure in the brain: Implications for synaptic dysfunction and cognitive decline. Curr. Med. Chem..

[19] Pfrieger F.W. (2003). Role of cholesterol in synapse formation and function. Biochim. Biophys. Acta.

[20] Segatto M., Manduca A., Lecis C., Rosso P., Jozwiak A., Swiezewska E., Moreno S., Trezza V., Pallottini V. (2014). Simvastatin treatment highlights a new role for the isoprenoid/cholesterol biosynthetic pathway in the modulation of emotional reactivity and cognitive performance in rats. Neuropsychopharmacology.

[21] Moutinho M., Nunes M.J., Rodrigues E. (2017). The mevalonate pathway in neurons: It’s not just about cholesterol. Exp. Cell Res..

[22] Mazzucchelli C., Brambilla R. (2000). Ras-related and MAPK signalling in neuronal plasticity and memory formation. Cell. Mol. Life Sci..

[23] Lingor P., Teusch N., Schwarz K., Mueller R., Mack H., Bähr M., Mueller B.K. (2007). Inhibition of Rho kinase (ROCK) increases neurite outgrowth on chondroitin sulphate proteoglycan in vitro and axonal regeneration in the adult optic nerve in vivo. J. Neurochem..

[24] Cartocci V., Segatto M., Di Tunno I., Leone S., Pfrieger F.W., Pallottini V. (2016). Modulation of the Isoprenoid/Cholesterol Biosynthetic Pathway During Neuronal Differentiation In Vitro. J. Cell. Biochem..

[25] Roy A., Jana M., Kundu M., Corbett G.T., Rangaswamy S.B., Mishra R.K., Luan C.H., Gonzalez F.J., Pahan K. (2015). HMG-CoA Reductase Inhibitors Bind to PPARα to Upregulate Neurotrophin Expression in the Brain and Improve Memory in Mice. Cell Metab..

[26] McCauslin C.S., Heath V., Colangelo A.M., Malik R., Lee S., Mallei A., Mocchetti I., Johnson P.F. (2006). CAAT/enhancer-binding protein delta and cAMP-response element-binding protein mediate inducible expression of the nerve growth factor gene in the central nervous system. J. Biol. Chem..

[27] Towler M.C., Hardie D.G. (2007). AMP-activated protein kinase in metabolic control and insulin signaling. Circ. Res..

[28] Janssens V., Goris J. (2001). Protein phosphatase 2A: A highly regulated family of serine/threonine phosphatases implicated in cell growth and signalling. Biochem. J..

[29] Espenshade P.J., Hughes A.L. (2007). Regulation of sterol synthesis in eukaryotes. Annu. Rev. Genet..

[30] Horton J.D., Goldstein J.L., Brown M.S. (2002). SREBPs: Activators of the complete program of cholesterol and fatty acid synthesis in the liver. J. Clin. Investig..

[31] Segatto M., Trapani L., Lecis C., Pallottini V. (2012). Regulation of cholesterol biosynthetic pathway in different regions of the rat central nervous system. Acta Physiol..

[32] Lowry O.H., Rosebrough N.J., Farr A.L., Randall R.J. (1951). Protein measurement with the Folin phenol reagent. J. Biol. Chem..

[33] McCaffrey K.A., Jones B., Mabrey N., Weiss B., Swan S.H., Patisaul H.B. (2013). Sex specific impact of perinatal bisphenol A (BPA) exposure over a range of orally administered doses on rat hypothalamic sexual differentiation. Neurotoxicology.

[34] Furtado R.H.M., Giugliano R.P. (2020). What Lessons Have We Learned and What Remains to be Clarified for PCSK9 Inhibitors? A Review of FOURIER and ODYSSEY Outcomes Trials. Cardiol. Ther..

[35] Kundakovic M., Gudsnuk K., Herbstman J.B., Tang D., Perera F.P., Champagne F.A. (2015). DNA methylation of BDNF as a biomarker of early-life adversity. Proc. Natl. Acad. Sci. USA.

[36] Tonini C., Schiavi S., Macca F., Segatto M., Trezza V., Pallottini V. (2020). Long-lasting impact of perinatal dietary supplementation of omega 3 fatty acids on mevalonate pathway: Potential role on neuron trophism in male offspring hippocampal formation. Nutr. Neurosci..

[37] Wang C., Li Z., Han H., Luo G., Zhou B., Wang S., Wang J. (2016). Impairment of object recognition memory by maternal bisphenol A exposure is associated with inhibition of Akt and ERK/CREB/BDNF pathway in the male offspring hippocampus. Toxicology.

[38] Godfrey K.M., Barker D.J. (1995). Maternal nutrition in relation to fetal and placental growth. Eur. J. Obstet. Gynecol. Reprod. Biol..

[39] Rice D., Barone S. (2000). Critical periods of vulnerability for the developing nervous system: Evidence from humans and animal models. Environ. Health Perspect..

[40] Thelen K.M., Falkai P., Bayer T.A., Lütjohann D. (2006). Cholesterol synthesis rate in human hippocampus declines with aging. Neurosci. Lett..

[41] Hirose M., Ishizaki T., Watanabe N., Uehata M., Kranenburg O., Moolenaar W.H., Matsumura F., Maekawa M., Bito H., Narumiya S. (1998). Molecular dissection of the Rho-associated protein kinase (p160ROCK)-regulated neurite remodeling in neuroblastoma N1E-115 cells. J. Cell Biol..

[42] Fracassi A., Marangoni M., Rosso P., Pallottini V., Fioramonti M., Siteni S., Segatto M. (2019). Statins and the Brain: More than Lipid Lowering Agents?. Curr. Neuropharmacol..

[43] Caserta M., Ben-Soussan T.D., Vetriani V., Venditti S., Verdone L. (2019). Influence of Quadrato Motor Training on Salivary proNGF and proBDNF. Front. Neurosci.

[44] Segatto M., Fico E., Gharbiya M., Rosso P., Carito V., Tirassa P., Plateroti R., Lambiase A. (2019). VEGF inhibition alters neurotrophin signalling pathways and induces caspase-3 activation and autophagy in rabbit retina. J. Cell. Physiol..

[45] Warita K., Mitsuhashi T., Ohta K., Suzuki S., Hoshi N., Miki T., Takeuchi Y. (2013). In vitro evaluation of gene expression changes for gonadotropin-releasing hormone 1, brain-derived neurotrophic factor and neurotrophic tyrosine kinase, receptor, type 2, in response to bisphenol A treatment. Congenit. Anom..

[46] Meyer M., Matsuoka I., Wetmore C., Olson L., Thoenen H. (1992). Enhanced synthesis of brain-derived neurotrophic factor in the lesioned peripheral nerve: Different mechanisms are responsible for the regulation of BDNF and NGF mRNA. J. Cell Biol..

[47] Duan L., Chen B.Y., Sun X.L., Luo Z.J., Rao Z.R., Wang J.J., Chen L.W. (2013). LPS-induced proNGF synthesis and release in the N9 and BV2 microglial cells: A new pathway underling microglial toxicity in neuroinflammation. PLoS ONE.

[48] Noga O., Hanf G., Görges D., Dinh Q.T., Groneberg D.A., Suttorp N., Kunkel G. (2005). Regulation of NGF and BDNF by dexamethasone and theophylline in human peripheral eosinophils in allergics and non-allergics. Regul. Pept..

[49] Lannigan D.A. (2003). Estrogen receptor phosphorylation. Steroids.

[50] Duplessis T.T., Williams C.C., Hill S.M., Rowan B.G. (2011). Phosphorylation of Estrogen Receptor α at serine 118 directs recruitment of promoter complexes and gene-specific transcription. Endocrinology.

[51] Bolli A., Galluzzo P., Ascenzi P., Del Pozzo G., Manco I., Vietri M.T., Mita L., Altucci L., Mita D.G., Marino M. (2008). Laccase treatment impairs bisphenol A-induced cancer cell proliferation affecting estrogen receptor alpha-dependent rapid signals. IUBMB Life.

[52] Marino M., Pellegrini M., La Rosa P., Acconcia F. (2012). Susceptibility of estrogen receptor rapid responses to xenoestrogens: *Physiological outcomes*. Steroids.

